# Surgeon-General William Burns Beatson

**Published:** 1911-05-13

**Authors:** 


Surgeon-General William Burns Beatson, of the Indian Medical Service, has died, in his eighty-seventh year, at Eastbourne. He studied at Guy's and St. Andrew's University, where he graduated M.D. in 1852, qualified in 1846, and as ship's doctor made three voyages round the Cape of Good Hope. He then became assistant surgeon in the East India Company's Army in India for twenty-five years. His appointments included those of superintendent of the lunatic asylum, medical officer to the Central Gaol, and Principal of the School of Medicine at Nagpur. He was the author of "The Indian Medical Service, Past and Present," of which a revised edition has been issued. He gained the F.R.C.S. in 1867, and was a member of the Royal Asiatic Society.

